# Band importance for speech-in-speech recognition

**DOI:** 10.1121/10.0005762

**Published:** 2021-08-02

**Authors:** Emily Buss, Adam Bosen

**Affiliations:** 1 Department of Otolaryngology/Head and Neck Surgery, University of North Carolina at Chapel Hill, Chapel Hill, North Carolina 27599, USA; 2 Center for Hearing Research, Boys Town National Research Hospital, Omaha, Nebraska 68131, USA ebuss@med.unc.edu, Adam.Bosen@boystown.org

## Abstract

Predicting masked speech perception typically relies on estimates of the spectral distribution of cues supporting recognition. Current methods for estimating band importance for speech-in-noise use filtered stimuli. These methods are not appropriate for speech-in-speech because filtering can modify stimulus features affecting auditory stream segregation. Here, band importance is estimated by quantifying the relationship between speech recognition accuracy for full-spectrum speech and the target-to-masker ratio by channel at the output of an auditory filterbank. Preliminary results provide support for this approach and indicate that frequencies below 2 kHz may contribute more to speech recognition in two-talker speech than in speech-shaped noise.

## 1. Introduction

Understanding how masking and audibility affect speech perception is critically important given the ubiquity of competing sound streams in natural listening environments (Hodgson *et al.*, 2007). The amount of acoustic information supporting speech perception is often characterized as the product of the audibility of each frequency band and the importance of the speech cues contained in that band, with additional factors related to presentation level and distortion associated with hearing loss (e.g., ANSI, 1997). This general approach has been tremendously successful at predicting speech recognition in quiet and in steady noise, with applications in both research and clinical contexts (e.g., Amlani *et al.*, 2002). However, simple models based on audibility as a function of frequency typically fail when the background contains spectro-temporally complex sounds like competing speech. This is problematic because many everyday listening environments contain multiple speech streams, and the ability to recognize speech under these conditions is important for successful communication (Phatak *et al.*, 2018).

One fundamental difference between speech-in-noise and speech-in-speech recognition could be the spectral distribution of cues necessary to support recognition. Whereas band importance for speech-in-noise recognition is thought to be determined predominantly by the spectral distribution of audible phonetic cues, speech-in-speech could also rely on access to cues supporting stream segregation and selective attention to the target. Listeners use differences in mean voice F0 and prosodic contour to segregate concurrent streams of speech (Assmann and Paschall, 1998; Calandruccio *et al.*, 2019; Darwin *et al.*, 2003), and prosodic information is preferentially represented at low frequencies (Grant and Walden, 1996). This could result in greater reliance on low-frequency cues when listening to speech-in-speech than speech-in-noise. Bandwidth requirements are also greater for speech-in-speech recognition compared to speech-in-noise (Best *et al.*, 2019), which could reflect flatter, more uniform band importance functions (BIFs) for speech-in-speech than speech-in-noise recognition.

The classic approach for measuring BIFs for speech-in-noise entails characterizing recognition in speech-shaped noise for a range of target-to-masker ratios (TMRs) and a range of low- or high-pass filter cutoffs (French and Steinberg, 1947; Studebaker and Sherbecoe, 1991). The resulting BIFs differ for different stimuli and as a function of listening context (ANSI, 1997; DePaolis *et al.*, 1996), but they are typically bandpass with a peak in the region of 1–3 kHz. Band importance has also been estimated using narrow bands of speech distributed across frequency (Bosen and Chatterjee, 2016; Healy *et al.*, 2013). The BIFs estimated using narrowband speech differ from those based on low- and high-pass speech, notably in terms of microstructure, which is hypothesized to reflect the formant structure of the speech sample (e.g., Healy *et al.*, 2013). Methods relying on filtered speech are not appropriate for measuring BIFs for speech-in-speech recognition because filtering can modify masker intelligibility and the perceptual similarity of the target and masker, both factors that play an important role in recognition of speech in a speech masker [reviewed by Calandruccio *et al.* (2017)]. In other words, filtering speech could change the relative contributions of energetic and informational masking.

This report describes an approach for estimating BIFs by evaluating the relationship between audibility of speech cues in restricted frequency bands and correctness of listener response across stimulus samples of unmodified, full-bandwidth speech-in-noise and speech-in-speech. A preliminary test of this approach was undertaken using data reported by Buss *et al.* (2020) for adults with normal hearing. That study measured word recognition in speech-shaped noise and in two-talker speech, using either reproducible target-plus-masker stimuli (described as frozen) or randomly paired stimuli. When stimuli were frozen, performance was consistent both within and between listeners—some stimuli were reliably easier than others. In the noise masker, these stimulus differences were attributed to differences across target words, but in the two-talker masker, there was evidence that the combination of the target and masker sample affected recognition. A simple model of speech glimpsing based on the work of Cooke (2006) provided support for the idea that differences in target audibility across stimuli affected recognition, with comparable effects in both the noise masker and the speech masker. The next logical question is whether the spectral distribution of audible speech cues affects performance similarly for speech-in-noise and speech-in-speech.

The present report uses the data of Buss *et al.* (2020) to evaluate the feasibility of estimating BIFs using unmodified, full-bandwidth stimuli. We predicted that the BIFs for speech-in-noise obtained using these methods would resemble published functions estimated using low-pass and high-pass filtered speech (Fletcher and Galt, 1950; French and Steinberg, 1947). It was unclear what to expect for speech-in-speech recognition, but reliance on segregation cues related to voice F0 could result in greater low-frequency emphasis in the BIF, and reliance on cues distributed across a wide bandwidth of speech could indicate a less peaky, broader BIF.

## 2. Methods

Buss *et al.* (2020) measured word recognition in a four-alternative forced-choice (4AFC) task. Targets and response alternatives were drawn from a set of 30 disyllabic words. Maskers were 30 samples of either speech-shaped noise or two-talker speech, each 2.8 s in duration. Different female talkers produced the target and masker speech. Stimuli were presented at 65 dB SPL. The first stage of testing entailed estimating speech reception thresholds, and the second stage evaluated performance at a fixed TMR corresponding to approximately 65% correct as determined for each listener. The masker sample paired with each target was either *random*, selected from among the 30 alternatives each time the stimulus was heard, or it was *frozen*, meaning that each target was paired with the same masker sample each time it was presented. Each listener completed two blocks of fixed-TMR testing in each of four conditions: two target-to-masker pairings (random and frozen) for each of two maskers (speech-shaped noise and two-talker speech).

The fixed-TMR data evaluated here were provided by 24 adults with normal hearing (18–41 years), all native speakers of American English. This dataset includes 1440 trials in each condition (24 listeners × 2 blocks × 30 trials). Across listeners, TMRs used for fixed-level testing were −25 to –10 dB [mean (M) = –20 dB] for the two-talker masker and −16 to –10 dB (M = –13 dB) for the speech-shaped noise, resulting in mean performance near 65% correct. Buss *et al.* (2020) evaluated the glimpsing opportunities available for these stimuli and argued that listeners required more audible glimpses to recognize speech-in-speech than speech-in-noise. This result indicates that the two-talker speech masker was associated with informational masking even though thresholds were lower than those in the speech-shaped noise masker.

Weights comprising the BIF were computed by passing target and masker samples through a bank of 35 gammatone filters with center frequencies between 125 and 12 000 Hz, each separated by approximately one equivalent rectangular bandwidth (Moore and Glasberg, 1983). The TMR was calculated for each frequency band as the mean difference between target and masker envelope amplitude on a log scale over the duration of each target word. The relationship between TMR in each frequency band and response accuracy provides an estimate of the weight of each band. This relationship was evaluated in two ways. For the frozen stimuli, the mean proportion correct across listeners was computed for each of the 30 target words, and the Pearson correlation with TMR was computed on the logit-transformed proportion correct. A similar approach has been used previously to evaluate effects of audibility for recognition of speech masked by narrow bands of noise with level jitter (e.g., Calandruccio and Doherty, 2007; Doherty and Turner, 1996). This correlation-based approach cannot be used for the *random* condition, where the target-to-masker pairings differ across listeners and between blocks of trials within listeners. A second type of analysis was therefore undertaken, using logistic regression to fit trial-by-trial listener responses, with random intercepts for each listener and stimulus sample [as in Bosen and Chatterjee (2016)]. Separate models were fitted for each frequency band. In this approach, band importance is reflected in the log odds of a correct response by TMR. In both approaches for quantifying weights, associations strong enough to reach statistical significance indicate bands that substantially contribute to speech intelligibility.

**Fig. 1.**
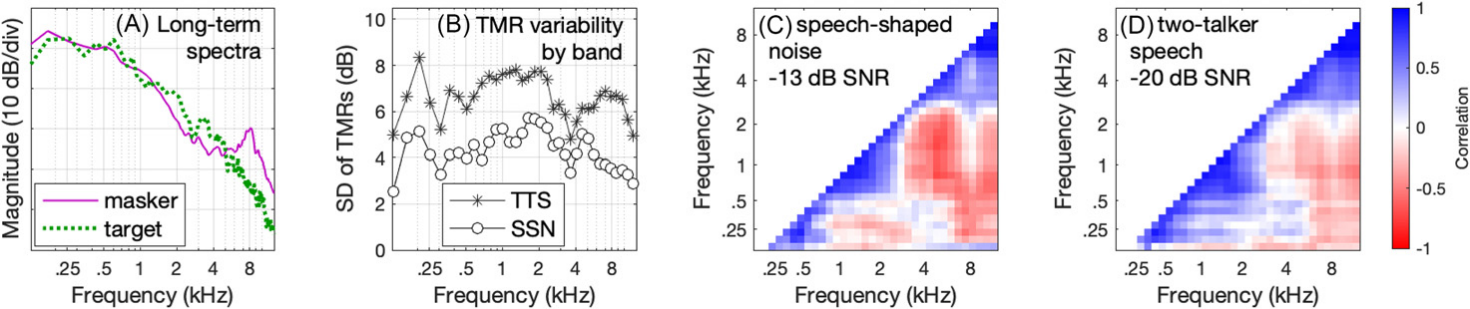
Acoustic features of the stimuli. (A) Long-term magnitude spectra of the 30 target words and samples of the speech-shaped noise masker. Two-talker speech is not shown because the two maskers had functionally identical long-term magnitude spectra. (B) Standard deviation of the mean TMRs in each frequency band for frozen stimuli, plotted separately for the two-talker speech (TTS) and speech-shaped noise (SSN) maskers. (C) Correlations across frequency bands for the TMRs associated with each of 30 frozen stimuli, with targets presented in the speech-shaped noise masker at −13 dB TMR (the group-mean average). Shading reflects the value of the correlation for each pair of bands as defined in the legend at the far right of the figure. The diagonal is omitted to highlight pairwise correlation as a function of band separation in Hz. (D) Correlations across frequency bands for targets presented in the two-talker speech masker at −20 dB TMR (the group-mean average).

Figure 1 illustrates several important features of the stimuli used by Buss *et al.* (2020). Figure 1(A) shows the long-term magnitude spectra of the 30 targets and masker samples, plotted as a function of frequency. The two maskers are not shown separately because their magnitude spectra are functionally identical. There is relatively more target energy between 1.3 and 5 kHz and more masker energy above 5 kHz. Mismatches in the long-term magnitude spectra of target and masker stimuli can impact the BIF by virtue of differences in mean cue audibility (e.g., Pollack, 1948). Although the long-term magnitude spectra are essentially the same for the speech-shaped noise and two-talker speech maskers, temporal fluctuations characteristic of two-talker speech would provide epochs of high audibility across the spectrum, which could also affect band importance.

Figure 1(B) shows the standard deviation of the mean TMRs for the 30 frozen stimulus samples as a function of frequency. The TMR was more variable across samples for the two-talker speech masker than the speech-shaped noise masker, with mean values of 6.6 and 4.3 dB, respectively. Variability in TMRs differed across frequency bands, and these functions were approximately parallel for the two maskers, including peaks around 200 Hz and dips around 3500 Hz. Repeating this analysis for stimuli in the random condition (not shown) produced very similar results, with the caveat that the standard deviation across samples in each masker differed less as a function of frequency due to the larger number of stimulus samples. Based on this figure, variability in TMR across bands would not be expected to differentially affect BIFs in the two-talker speech and speech-shaped noise maskers.

Figures 1(C) and 1(D) show the intercorrelation between TMRs across frequency bands for targets presented at the group-mean average TMR (–13 dB for speech-shaped noise and –20 dB for two-talker speech), plotted separately for the two maskers. For these panels, cell shading reflects the magnitude of the correlation as defined in the legend at the far right. Correlations tended to be strongly positive for neighboring bands, with a mean value of *r* = 0.88 for both maskers. Negative correlations were observed for some pairs of bands, with greater evidence of negative correlation in the speech-shaped noise masker. For example, the strongest negative correlations for the speech-shaped noise stimulus were between bands in the region of 4.5–5.0 kHz and those in the region of 0.7–2.0 kHz, with values of *r* = −0.72 to −0.46. In contrast, associated correlations for the two-talker speech masker were *r* = –0.36 to −0.07. The stimuli used to generate correlations shown in Figs. 1(C) and 1(D) differed with respect to overall TMR, but a similar pattern of results is observed when stimuli are evaluated at the overall TMRs for the poorest-performing listener (–10 and −11 dB). One explanation for the reduced evidence of negative correlations across bands in the two-talker speech masker is related to the presence of multiple simultaneous voices. Whereas the spectral distribution of energy is limited by the configuration of the vocal tract in a single talker, no such restriction is imposed when there are multiple talkers. These stimulus features will be revisited when interpreting BIF data.

Stimuli, data, and matlab scripts for computing weights are available online (OSF, 2021).

## 3. Results

**Fig. 2.**
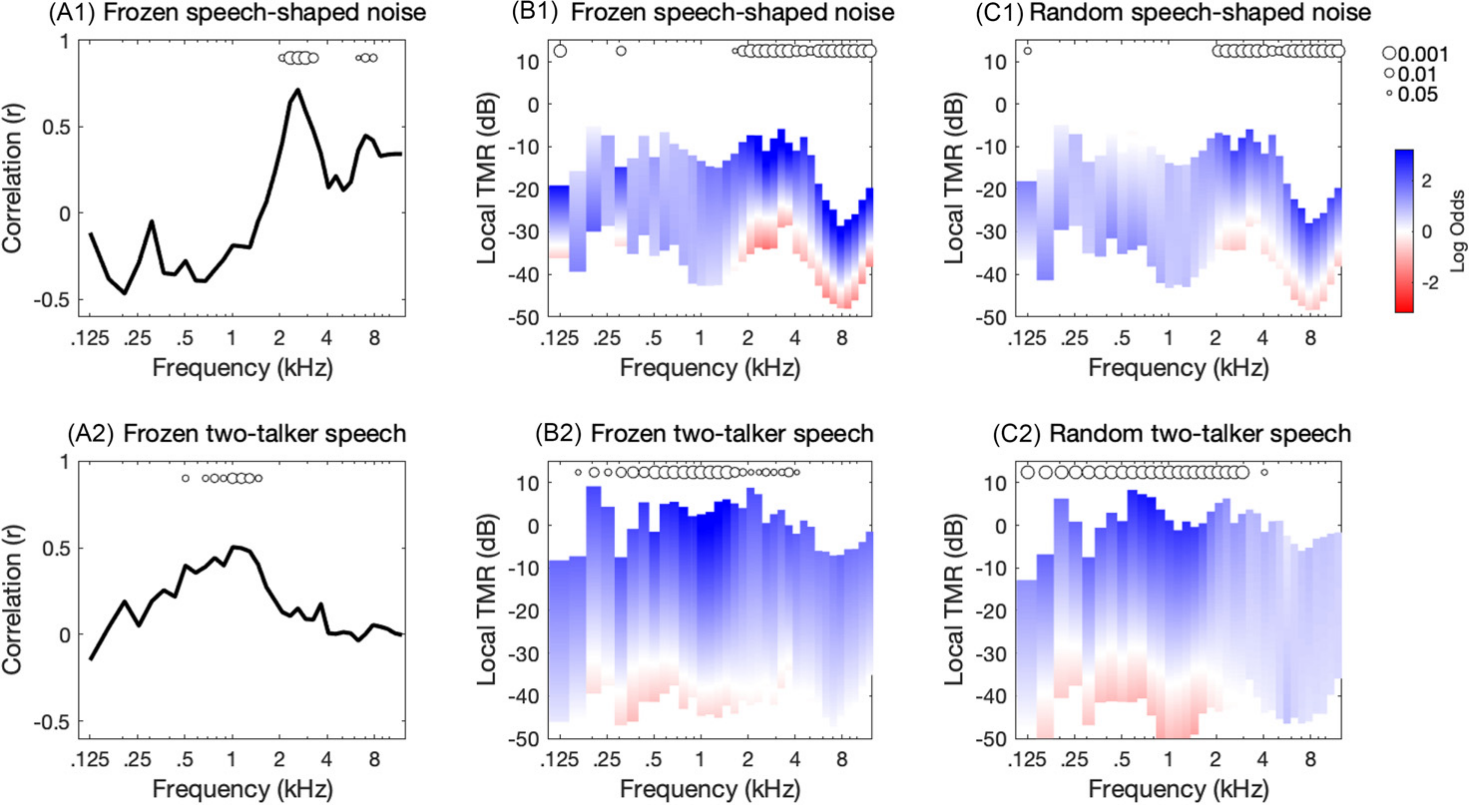
Band importance for word recognition plotted as a function of frequency, shown separately for data collected in speech-shaped noise (top row) and two-talker speech (bottom row). [(A1) and (A2)] Pearson correlation between TMR by frequency for frozen target-plus-masker samples and logit-transformed mean proportion correct. [(B1) and (B2)] Log odds of a correct response based on logistic regression for the frozen condition. Bar shading indicates the log odds as defined in the legend at the far right. The vertical extent of each bar reflects the range of TMRs observed across trials. [(C1) and (C3)] Log odds of correct response for the random condition. Circles at the top of each panel indicate the significance of the association between listener responses and TMRs for each frequency band as defined in the legend at the far right.

The relationship between the TMR by frequency band and correct recognition is shown in Fig. 2, with results for the speech-shaped noise masker in the top row of panels and those for the two-talker speech masker in the bottom row. Circles at the top of each panel indicate significant positive associations between mean TMR and correct recognition as defined in the legend at the far right of the figure.

The correlation between TMR by frequency band and the logit-transformed mean proportion correct across listeners is considered first. For the speech-shaped noise masker [Fig. 2(A1)], positive weights were observed at and above approximately 1.5 kHz, with a peak at 2.6 kHz. Non-significant negative weights for channels below 1.5 kHz suggest a reduced contribution to recognition, but they should not be interpreted as indicating that target audibility in these frequency regions hurts performance. Since target stimuli were root mean square (rms) normalized, samples with greater low-frequency energy would tend to have less high-frequency energy, particularly in light of the negative correlations between TMRs at 4.5–5.0 kHz and those at 0.7–2.0 kHz. For the two-talker speech masker [Fig. 2(A2)], weights are positive between about 150 Hz and 4 kHz, with a broad peak at 1 kHz. This finding is consistent with the idea that speech-in-speech recognition relies on a broader range of frequencies, including low frequencies, compared to speech-in-noise recognition.

Results obtained using logistic regression are generally consistent with those based on correlation. Figures 2(B1) and 2(B2) show results for the frozen conditions, the same data evaluated using the correlation methods. Figures 2(C1) and 2(C2) show results for the random conditions. Bars in these panels indicate the log odds associated with the regression fit for each frequency band as defined in the legend at the far right of the figure, and the vertical extent of each bar reflects the range of TMRs observed for each frequency band. Bars with more marked changes in shading indicate greater reliance on TMR for recognition. For the speech-shaped noise masker, an association between TMR by frequency and listener responses was predominantly observed at and above 1.6 kHz [Figs. 2(B1) and 2(C1)], but for the two-talker speech masker, associations were observed at and below 2.6 kHz [Figs. 2(B2) and 2(C2)]. The finding of similar BIFs for the frozen conditions using the two analysis approaches (correlation and logistic regression) indicates that the method used to demonstrate the relationship between TMR by frequency and recognition is not critical. The finding of similar BIFs for the frozen and random conditions indicates that the results observed can be replicated across datasets, namely, that word recognition in the two-talker masker appears to rely on lower-frequency channels and a broader spectral distribution of information than recognition in the speech-shaped noise masker.

Plots showing results of the logistic regression analysis illustrate two additional features of interest. First, variability in the vertical placement of bars across frequency reflect differences in the range of TMRs across frequency bands. For example, the median TMR by frequency for the frozen speech-shaped noise condition was −20 dB at 3.3 kHz and −41 dB at 7.8 kHz. These differences reflect the differences in long-term magnitude spectra for the targets and masker [Fig. 1(A)]. Second, the vertical extent of bars is smaller for data taken in the speech-shaped noise compared to the two-talker speech masker. Whereas TMRs span a range of 24 dB on average for the speech-shaped noise, that range is 42 dB for the two-talker speech. This reflects the marked envelope fluctuation in the speech masker, resulting in greater variability in TMR [Fig. 1(B)]. Recall that TMRs calculated for each band were computed by taking the mean difference between the target level and the masker level. A positive log odds ratio at a low mean TMR therefore does not necessarily indicate that listeners made use of cues at that TMR; it could also reflect benefit from brief epochs when the TMR was higher than the mean.

## 4. Discussion

The analyses described in this report estimated BIFs for masked speech recognition and evaluated whether BIFs differ for speech-in-speech as compared to speech-in-noise. Data were taken from a published study on forced-choice word recognition in adults with normal hearing. Stimuli were passed through a gammatone filterbank, and the mean TMR by frequency band was computed for each stimulus. Two methods were used for deriving BIFs from these data, one based on correlation and the other based on logistic regression. Both methods indicate differences in the BIFs for speech-in-speech and speech-in-noise. The BIFs for speech-in-noise recognition indicate the greatest reliance on cues in the region of 2.6 kHz. In contrast, those for speech-in-speech recognition indicate reliance on a broader distribution of low-frequency cues, including 0.5–1.5 kHz.

Using full-bandwidth stimuli to estimate weights has the advantage of preserving the perceptual features known to affect performance in multi-talker environments, but it is not clear whether these methods produce similar results compared to more traditional methods for evaluating BIFs. Although correlation has been used previously to evaluate the effects of level jitter applied to bands of masking noise (e.g., Doherty and Turner, 1996), there is no precedent for using this approach to characterize BIFs for unmodified stimuli, where variation in TMR by frequency is due to inherent modulation rather than experimental manipulation. Confidence in these methods would be bolstered if results obtained for speech-in-noise recognition resembled those observed previously using more traditional methods. Several studies report BIFs with peaks in the region of 2–3 kHz for monosyllabic word recognition (Black, 1959; French and Steinberg, 1947). One caveat is that results differ markedly across studies (DePaolis *et al.*, 1996), and the phonetic content of target words and response alternatives in a closed-set task can affect band importance (Duggirala *et al.*, 1988). Therefore, while results are broadly similar to those observed previously for words in noise, a rigorous comparison of BIFs for speech-in-noise across estimation methods would require data obtained using the same stimuli and recognition task.

For speech-in-noise recognition, mismatches in target and masker spectra can influence the BIF. For example, Pollack (1948) measured word recognition in a white-noise masker at a range of low- and high-pass filter cutoffs and at a range of TMRs. They observed greater relative contributions of high-frequency information at higher TMRs, an effect that they attributed to increasing audible high-frequency bandwidth with increasing TMR for speech in a white-noise masker. Recall that the TMR used for fixed-level testing by Buss *et al.* (2020) was selected to produce approximately 65% correct performance, and the group-mean TMR differed for the speech-shaped noise (–13 dB) and two-talker speech (–20 dB) conditions. This raises the question of whether differences in the long-term magnitude spectra of the target and masker [Fig. 1(A)] could differentially affect BIFs estimated for the two maskers. The data of Pollack (1948) would predict that weights at a lower TMR should be reduced in frequency regions associated with relatively more masking. For the stimuli of Buss *et al.* (2020), greater high-frequency masking is associated with frequencies ≥5 kHz. This would predict reduced weights at and above 5 kHz for the two-talker speech masker (tested at a lower TMR) relative to the speech-shaped noise masker (tested at a higher TMR), but it would not account for the reduced weights at mid frequencies (e.g., 2–4 kHz). Further, due to the temporal fluctuations in the speech masker, greater masking on average at and above 5 kHz would not be expected to preclude transient audibility in the two-talker speech masker even at a low TMRs.

This report provides preliminary data on techniques for deriving BIFs from full-bandwidth stimuli, but there are several important caveats. The data used to evaluate these techniques were obtained with a limited stimulus set of only 30 targets and 30 masker samples of each type (speech-shaped noise and two-talker speech). Confidence in the results is bolstered by the use of identical targets and matched long-term magnitude spectra in the two masker conditions as well as similarities in the BIFs based on data from the frozen and random conditions. However, it is still unclear whether the masker effects observed here are representative of those associated with other corpora or experimental conditions. Another limitation is ambiguity regarding the factors responsible for differences in the BIFs observed for noise and speech maskers. For example, cues supporting auditory stream segregation could be responsible for the greater importance of low-frequency cues for speech-in-speech recognition, but we cannot rule out frequency-specific factors related to audibility, forward masking, or spread of excitation. Finally, it is well known that speech cues combine nonlinearly across frequency (Humes and Kidd, 2016; Pollack, 1948; Warren *et al.*, 2005). The BIFs reported here do not capture redundancy or synergy of information, which could differ across masker contexts.

Characterizing differences in the BIF for speech recognition in different masker contexts, including background speech, could be critical to improving our ability to predict speech recognition under different listening conditions. More broadly applicable models of speech recognition would have a wide range of practical applications, including as tools for patient counselling and fitting hearing aids (Amlani *et al.*, 2002). There is also growing interest in modeling the peripheral effects associated with speech-in-speech recognition in the context of laboratory research (e.g.,Vicente *et al.*, 2020). Characterizing BIFs for speech-in-speech recognition could be particularly effective as an approach for differentiating effects of audibility from central factors because individual differences in peripheral encoding can obscure effects related to cognition and language ability, and vice versa (Humes, 2007; van Rooij and Plomp, 1991). The success of these efforts could rely on the accuracy of BIFs used in models of speech recognition, which are currently based on data obtained in noise. The results presented here suggest that BIFs can be estimated for speech-in-speech and that they differ compared to those for speech-in-noise.
